# North Carolina's Amended Dental Law

**Published:** 1887-10

**Authors:** 


### Editorial, Etc.

---

NORTH CAROLINA'S AMENDED DENTAL LAW.—Below will be found the text of the amended dental law of North Carolina, as passed by the Legislature.

*The General Assembly of North Carolina do enact:*

SECTION 1. That the section 3148 of the Code of North Carolina, being chapter xxxiv of vol. ii, be stricken out and the following inserted in lieu thereof:

Hereafter no person shall commence the practice of dentistry who has not obtained a certificate from a Board of Examiners duly authorized and appointed in accordance with section 3149, and that part of chapter xxxiv which related to dentistry, which certificate shall be registered in the office of the clerk of the superior court of the county in which such person proposes to practice, for which the clerk shall receive a fee of fifty cents. Provided, this section shall not apply to persons holding a diploma from a chartered dental institute.

SEC. 2. Any failure, neglect, or refusal on the part of any person holding such certificate to register the same, as above directed, for a period of six months, shall work a forfeiture of the certificate, aud no certificate, when once forfeited, shall be restored, except upon the payment to said Board of Examiners of the sum of twenty-five dollars as a penalty for such neglect, failure, or refusal.

SEC. 3. In order to provide means for carrying out and maintaining the provisions of this act, the said Board of Examiners may charge a fee of ten dollars for each person applying for a certificate, which in no case shall be returned; and the funds so derived shall be placed in the hands of the secretary, to be used in defraying the necessary expenses in conducting the meetings of said board, and under no circumstances shall any part of such expenses come out of the treasury of the State.

SEC. 4. Within six months from the time this act takes effect, it shall be the duty of every person who is at that time lawfully engaged in the practice of dentistry in this State to cause his or her name, residence, date of diploma, or license and date of commencing of the practice of dentistry, to be registered with the Secretary of the State Board of Dental Examiners authorized and appointed as aforesaid, in a book kept for that purpose. The statement of every such person shall be verified on oath before a notary public or justice of the peace in such manner as may be prescribed by the said Board of Examiners, which shall provide upon application blanks for this purpose. It shall be the duty of the secretary of the board to furnish the clerk of the superior court of each county a certified list of the names of all persons in said county who have registered according to the provisions of the act; and it shall be the duty of such clerk to register such names in a book kept for that purpose upon the payment to him of a fee of fifty cents. Any person thus registered can practice in one or more counties upon filling in such county or counties a duly certified transcript of such registration. All persons now practicing who shall fail to register according to the provision of this act within the time prescribed, and who shall offer to practice dentistry, shall be deemed guilty of a misdemeanor, and upon conviction shall be fined not more than fifty dollars nor less than twenty-five dollars for each offense. Any person who shall knowingly and falsely claim or pretend to have or hold a certificate of proficiency granted by said Board of Examiners shall be guilty of a misdemeanor, and upon conviction shall be fined not more than fifty dollars nor less than twenty-five dollars for each offense. All fines and penalties so recovered shall be appropriated to the school fund of the county in which the same shall have been recovered.

SEC. 5. Nothing in this act shall be so construed as to prohibit any one from extracting teeth.

SEC. 6. That section 3156 of said chapter xxxiv is not intended to apply to this act.

SEC. 7. This act shall take effect from and after its ratification.

